# Clinical Nurse Specialists' Journey From Novice to Expert—A Systematic Mixed‐Method Review

**DOI:** 10.1111/scs.70300

**Published:** 2026-07-14

**Authors:** Sanna Marika Imeläinen, Tarja Anneli Kvist, Mea Mirella Marjatta Wright, Krista Susanna Jokiniemi

**Affiliations:** ^1^ Department of Nursing Science University of Eastern Finland Kuopio Finland

**Keywords:** advanced practice nursing, clinical nurse specialist, expertise development, literature review, mixed‐method review, professional competence, staff development, systematic review

## Abstract

**Aims and Objectives:**

The mixed‐method review aimed to synthesize research on how clinical nurse specialists' expertise develops from novice to expert and how this progression can be supported.

**Methodological Design and Justification:**

The review was conducted in accordance with the Preferred Reporting Items for Systematic Reviews and Meta‐Analyses (PRISMA) and was registered on PROSPERO. A mixed‐methods approach was used to combine qualitative and quantitative data and enhance the robustness of the findings.

**Research Methods:**

Literature search was conducted in CINAHL, PubMed and Scopus in September 2024. The selection and quality assessment of the studies were performed using JBI critical appraisal tools. The synthesis was based on qualitative and quantitative studies, using Benner's ‘From Novice to Expert’ model as a framework.

**Ethical Issues and Approval:**

Ethical approval was not required for this study.

**Outcome Measures:**

The main outcomes were stages of expertise development and factors supporting progression.

**Results:**

Eleven articles were included for review. Becoming a clinical nurse specialist requires a basic nursing degree, clinical experience and a higher education degree. According to the results, expertise was seen to develop through three stages: novice, competent and expert, with the support of organizations, mentors, colleagues and multi‐professional collaboration. Pursuing continuing education while gaining experience is essential to carry on the journey towards expertise.

**Study Limitations:**

The study screening process may have introduced selection bias, potentially leading to the omission of relevant articles. Additionally, selected articles on other interventions, such as service development, were excluded. However, they could have provided further insights into development.

**Conclusions:**

This review shows that developing CNS expertise from novice to expert requires education, clinical experience, and continuous professional support. Strengthening structures and collaboration enhances patient care, while further research on support strategies and competencies is needed.

## 1 Introduction

According to the International Council of Nurses [1], a clinical nurse specialist (CNS) provides healthcare services based on advanced specialized expertise, particularly when treating complex and vulnerable patients or populations. CNSs work at an advanced level in nursing, engaging in broad and independent roles where increasingly specialized expertise is required. Alongside clinical expertise, CNSs have expertise in research, development, and service system management. These skills are crucial for implementing evidence‐based knowledge, adopting evidence‐based practices, and standardizing service system protocols. A CNS is a nurse who has a minimum of a master's degree specific to CNS practices [1, 2]. The role, competence requirements, and qualifications of CNSs have been thoroughly examined and identified in recent research [3, 4, 5]. Tools and checklists have been developed to describe and identify the required competencies [6, 7]. However, it is essential to acknowledge the variability of CNS roles across different countries [8].

The uniqueness of CNSs' work is seen to motivate further training [2]. When commencing a new position, CNSs are often novices. However, as they grow in their role and take on new responsibilities, their expertise begins to develop [9]. The development of competence is key to the development of expertise, which can be described as an ongoing process. In nursing, the evolution of competence begins during professional education and continues throughout the career. Competence develops with experience and training [10]. Furthermore, learning at work strengthens competence, so even after training as a CNS, years of practice are still required [11, 12].

The gap between theory and practice can be a driving force for continuous learning and encourage closer cooperation by creating structures involving individuals, teams, and organizations. Tools and structures will be provided to continue learning skills and competencies after graduation [13]. Moreover, continuing professional development among caregivers has been linked to increased job satisfaction [14]. Work‐integrated learning enables the integration of theory and practice, the development of competence, and preparation for working life. In addition, it supports the exchange of experiences between different healthcare professionals. Work‐integrated learning creates a link between education, healthcare organizations, and society to provide the best possible healthcare [15]. Social expectations and experiences impact professional attitudes and expertise development. Clinical experience and personal motivation, combined with self‐activity and maximizing personal potential, appear to have a decisive impact on the critical care provider's development towards professional excellence [16].

Furthermore, research and innovations are crucial for improving healthcare products, the quality, and the efficiency of services. Aligned with the Sustainable Development Goals (SDG), better health can be achieved through stronger collaboration for an equitable and resilient recovery in healthcare [17, 18]. One of the recommendations involves transforming the health workforce so that the skills of healthcare workers match the health needs of the population. To achieve this goal, working with the full potential is required [18]. In other words, there are multiple developmental opportunities for CNSs [19]. Utilizing the expertise of nursing staff benefits healthcare organizations and, most importantly, improves the quality of patient care [20].

Evidence‐based practices contribute to patient care and cost control [21]. Organizations can improve the effectiveness of care, ensure equal access to services, and improve service continuity through the development of evidence‐based expertise in healthcare and education. Hence, the development and utilization of CNS expertise is important. Policy changes will enhance the CNS practice. While there are various indications of increasing diversity in the role of CNS, there are also numerous challenges and accomplishments in the future [8].

According to Benner, challenges in the clinical environment drive healthcare personnel to develop nursing practices. Work‐integrated learning requires transparency and responsiveness from employees to improve practices. Skilled employees can use their previous knowledge proactively and intuitively, and opportunities to act in clinical situations with different alternatives increase. However, this applies to specific fields or types of nursing. Employees may be at different stages in terms of experience and skills when acting in different roles. For instance, expertise might be achieved in caring for adults, but this same stage does not necessarily apply to situations with children [22, 23].

The role of CNSs requires a shared understanding to enable meaningful dialogue about their professional development. This mutual comprehension is essential for ensuring their effective integration and utilization within healthcare organizations. Previous research has examined the role definitions and responsibilities of CNSs [2, 3, 4]. However, there remains a gap in knowledge regarding how CNS expertise develops and can be effectively supported. Nurses working at an advanced level, in autonomous and wide‐ranging roles, require continuous support to maintain competence and further refine their expertise [1].

**FIGURE 1:**
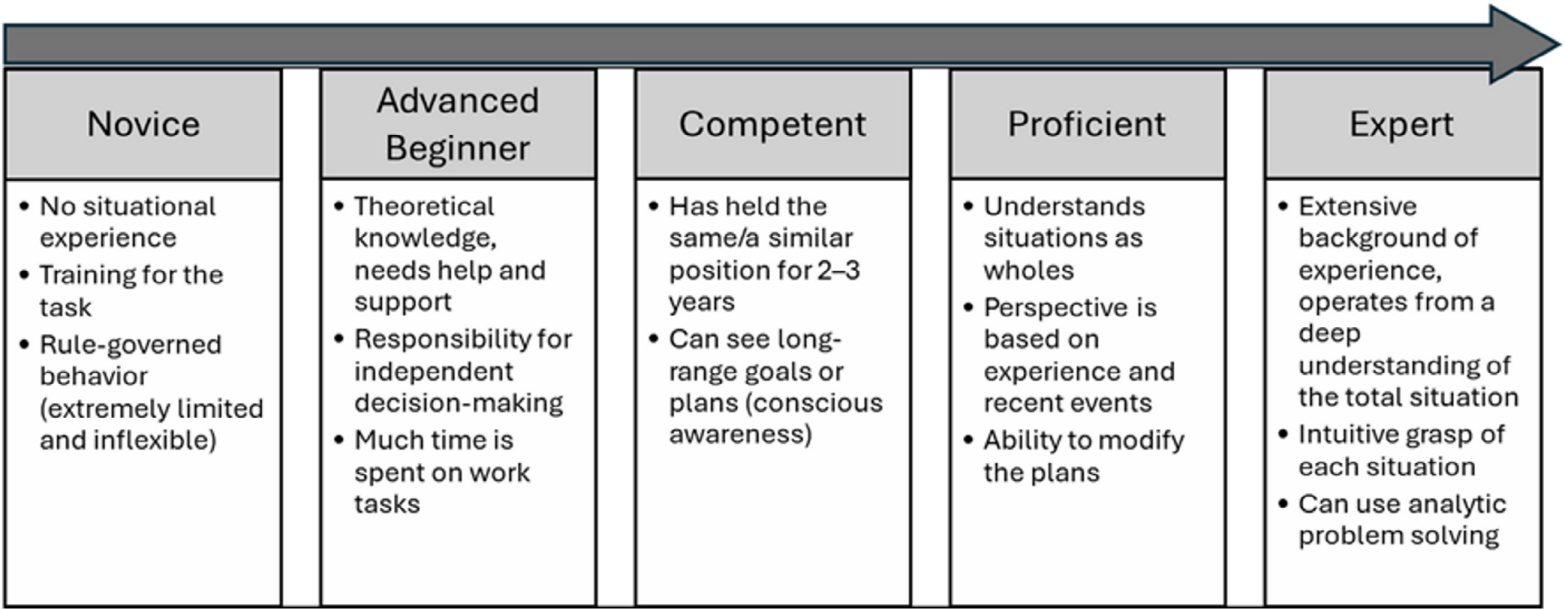
Stages in the development of expertise according to Benner's model [22, 23].

This study utilizes Benner's ‘From novice to expert’ model, derived from the Dreyfus model of skill acquisition, as its theoretical framework. Figure 1 illustrates this staged model of expertise development, showing the progression from novice to expert through increasing levels of experience, autonomy, and contextual understanding. According to Benner, expertise evolves through five distinct stages: novice, advanced beginner, competent, proficient, and expert [22, 23]. This systematic mixed‐method review synthesizes previous research on the development of CNS expertise and their journey from novice to expert.

The aim is to clarify how healthcare organizations and educational institutions can support the development of CNSs' expertise. A search of the databases CINAHL, PubMed, Scopus, the Cochrane Library, and Prospero revealed that no previous systematic reviews have been conducted on this topic. Mixed‐methods research combines qualitative and quantitative approaches to make study conclusions more comprehensive and robust, thereby contributing to the scientific literature [24]. The research questions of this study are: ‘How does the expertise of CNSs develop from novice to expert?’ and ‘How can the development of expertise be supported?’

## 2 Methods

### 2.1 Design

This systematic mixed‐methods review comprehensively synthesizes studies on the development of CNSs from novice to expert. This review followed the Joanna Briggs Institute (JBI) methodologies for mixed methods systematic reviews [25] and Preferred Reporting Items for Systematic Reviews and Meta‐Analyses (PRISMA) [26, 27]. It has been registered with PROSPERO (International Prospective Register of Systematic Reviews) with registration number CRD42022316457.

### 2.2 Search Methods

The search strategy was developed in collaboration with an information specialist and applied to the following databases: CINAHL, PubMed, and Scopus on December 27, 2021, and updated on September 6, 2024. The PICo method was used to comply with the topic and define research questions and keywords for data search. The identified PICo elements are shown in Table 1 [28]. The search phrase was (‘clinical nurse specialist*’ AND expertis* OR novice OR ‘advance* beginner’ OR competent OR proficient OR expert AND develop*). The search was limited to peer‐reviewed quantitative and qualitative research articles available in Finnish or English from 2012 to 2024.

**TABLE 1:** The PICo elements [28].

PICo‐method	The identified elements
*P* (Population)	Clinical nurse specialists and Clinical nurse specialist students, according to Benner's model (novice stage)
*I* (Phenomena of Interest)	Development of expertise (novice, advanced beginner, competent, proficient, expert)
*Co* (Context)	Healthcare

### 2.3 Study Screening and Data Extraction

The conceptual focus of this review was the development of expertise within the CNS role, guided by Benner's model. The target population included CNSs with a master's degree and, in line with Benner's framework, students preparing to enter the role [22, 23]. To ensure methodological rigour and relevance, only peer‐reviewed quantitative and qualitative studies meeting predefined inclusion and exclusion criteria were considered. Studies unrelated to the development of expertise, such as those focusing solely on role descriptions, administrative functions, or other advanced nursing roles, were excluded. Additionally, systematic reviews, conference abstracts, and non‐peer‐reviewed publications were omitted to maintain the quality and reliability of the synthesis.

**FIGURE 2:**
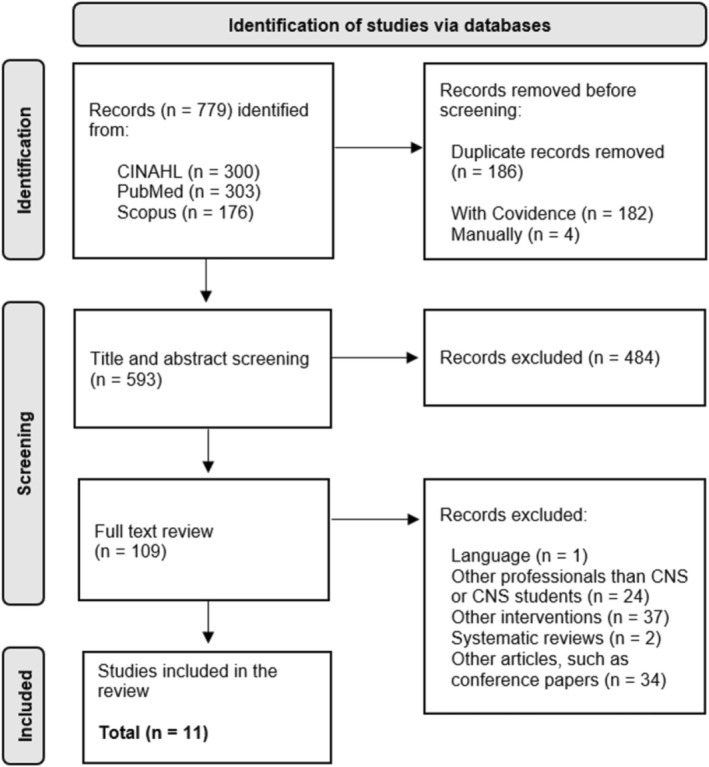
PRISMA 2020 flow diagram for systematic review according to Page et al. (2021) [26, 27].

The search produced a total of 779 articles. All articles found were imported into the Covidence program for systematic reviews [29]. Duplicate records were removed within Covidence (*n* = 182) before screening and manually (*n* = 4) during the review. Two authors (S.I. and M.W.) screened all articles independently. After screening titles and abstracts (*n* = 593), 109 articles were selected for full‐text review. Any disagreements regarding study selection at different stages were discussed to reach a consensus (K.J. and T.K.). The PRISMA flow chart illustrates the exclusion criteria for full‐text articles (Figure 2).

In total, 11 articles met the inclusion criteria. The PRISMA flow diagram for systematic reviews (Figure 2) illustrates the progression of studies through the different phases of the review process [26, 27]. Data extraction was conducted by one reviewer (S.I.) and discussed within the research team to ensure consistency and accuracy. Key study characteristics, including authorship, publication year, country, purpose or aims, population/sample size, and main findings, were tabulated (Table 2), and data were extracted accordingly. The extraction table preserves the integrity of the original studies before synthesis.

**TABLE 2:** Data extraction table.

Author(s), year, and country	Title	Aims/purpose	Population and sample size within the source of evidence	Methodology/methods	Outcomes and details	Role in results (Code)
Abel and Carter‐Templeton (2020) USA	Implementing a transition‐to‐practice program for novice clinical nurse specialists – A pilot project	The study aimed to determine the impact of a transition‐to‐practice program on job satisfaction among novice clinical nurse specialists (CNSs) in the US Air Force	A needs assessment was conducted on CNSs in the US Air Force (*n* = 30). Mentoring was conducted by newly graduated CNSs (*n* = 8)	The CNS Utilization Questionnaire via Qualtrics, an online questionnaire tool	Overall job satisfaction among novice clinical nurse specialists (CNSs) increased from 5.01 to 5.57 (scale 1–7) following program completion. The most notable improvements were observed in personal satisfaction (from 4.30 to 5.63) and satisfaction with professional support (from 5.28 to 6.00)	Codes: 1 and 5
Colwill et al. (2014) USA	Capture of knowledge work of clinical nurse specialists using a role tracking tool	The study aimed to quantify clinical nurse specialist (CNS) work and determine if competencies are associated with personal characteristics, priorities, and quality outcomes	Clinical nurse specialists (*n* = 14)	The investigator‐developed Role Tracker Tool (software) and a CNS questionnaire were used to collect baseline and monthly data for 5 months	Among 14 CNSs, the average age was 45 years, and CNS experience 5.6 years. ‘Quality’ and ‘clinical work’ were prioritized among six competencies, while ‘research’ ranked lowest. Clinical work consumed most of the time (1.9 h/day), and professional self‐development the least (0.4 h/day). Time allocation varied by specialty, experience, and perceived competence. Of nine quality initiatives, heart failure received the most attention (0.7 h/day), but time spent was not linked to improved outcomes. CNS priorities and quality‐related activities fluctuated over time	Codes: 1, 2, 3 and 5
Doody et al. (2017) Ireland	Activities of intellectual disability clinical nurse specialists in Ireland	The study aimed to identify the contribution of the Irish intellectual disability clinical nurse specialists (ID CNSs) to service delivery	Clinical nurse specialists (*n* = 32)	The research instrument used to collect the data was a 56‐item questionnaire that covered 8 sections developed based on the findings from focus group interviews and supporting literature	Findings identified that ID CNSs were active in all aspects of their roles as clinical specialists, educators, communicators, researchers, change agents, and leaders, thus supporting person‐centred care and improving service delivery. To meet changing healthcare demands, promote person‐centred care, and improve service delivery, the CNS role in ID should be developed and supported. The findings merited a further study on ID CNS role activity, possible variables influencing role activity, and team members' views	Codes: 5
Fallon et al. (2018) Ireland	Irish respiratory clinical nurse specialists'‐ experiences of their role	The study aimed to explore respiratory clinical nurse specialists' (CNSs') experiences of their role	Respiratory CNSs (*n* = 10) who worked across several health service regions in Ireland	CNSs were purposively sampled (*n* = 10), and data was collected by semi structured interviews	Intellectual disability (ID) clinical nurse specialists (CNSs) were actively engaged across all role domains, contributing to person‐centred care and enhanced service delivery. To address evolving healthcare needs, the ID CNS role requires further development and support. The findings highlight the need for continued research on role activity, influencing factors, and team perspectives	Codes: 2 and 5
Fulton et al. (2019) USA	Description of work processes used by clinical nurse specialists to improve patient outcomes	The purpose of the study was to identify common processes used by CNSs working in a variety of practice settings and specialties to advance nursing practice and achieve improved clinical outcomes	Clinical nurse specialists (*n* = 17) (8 and 9 in each focus group)	Qualitative descriptive methods were used; a purposeful sample of CNSs with completed system‐level projects participated in focus groups	CNSs engage in complex, often invisible articulation work at the intersection of people, technology, and organizations. Their practice is shaped by self‐agency, trust, and influence, which underpin core work processes. These findings clarify the CNS's leadership role and help explain the perceived invisibility and ambiguity of their practice	Codes: 1, 3 and 5
Joiner‐Rogers et al. (2019) USA	Using videoconferencing for verbal reports to improve clinical nurse specialist student performance	The purpose of the study was to determine the effectiveness of using videoconferencing for verbal reports as a learning strategy for improving clinical nurse specialist students' communication competencies and advanced practice decision‐making	Students in the final year of the AGCNS program (*N* = 28 over 2 cohorts)	Videoconferencing, using iPad minis issued to faculty and students, was used routinely for verbal reports on clinical cases to faculty, which included immediate faculty feedback.	Initially, student verbal reports were disorganized and inefficient. Regular videoconferencing practice reduced report duration from over 20 min to 3–5 min and improved accuracy. A structured, clinically relevant report template combined with immediate faculty feedback enhanced communication skills. Mobile technologies such as tablets and smartphones offer accessible tools to support this learning approach	Codes: 1 and 5
Kilpatrick et al. (2013) Canada	Practice patterns and perceived impact of clinical nurse specialist roles in Canada: Results of a national survey	The study aimed to gain a greater understanding of the structures, processes, and perceived outcomes of CNS roles in Canada	Overall, 776 questionnaires were returned electronically (*n* = 711; 91.6%) or by mail (*n* = 65; 8.4%)	A 50‐item self‐report questionnaire	The exact number of clinical nurse specialists (CNSs) in Canada remains unclear due to lack of title protection and inconsistent role identification. Among registry‐listed CNSs, the response rate was 33% (804/2431), with 608 confirming active CNS roles. Graduate‐prepared CNSs had a higher response rate of 60% (471/782). Practice patterns varied by specialty and were influenced by graduate‐level education. Structural and resource limitations hindered CNS role development	Codes: 5
Riordan et al. (2019) Ireland	‘Sink or Swim’: A qualitative study to understand how and why nurses adapt to support the implementation of integrated diabetes care	The aim was to understand how diabetes nurse specialists support the implementation of integrated care in a complex health system, including determinants of their behaviours	Of the 40 diabetes nurse specialists invited, 30 took part in total, in two focus groups (*n* = 8) and individual interviews (*n* = 23)	Semi‐structured focus groups and individual interviews were carried out with hospital and community diabetes nurse specialists across Ireland	Community nurse specialists faced role ambiguity upon appointment, often navigating their responsibilities independently. To establish their roles, they adapted to local contexts, addressed misconceptions, and built trust with general practitioners by aligning with practice norms. In the absence of multidisciplinary support, they worked autonomously and developed professional networks and ongoing education as alternative support structures. Workarounds were used to facilitate information flow between care settings due to the lack of shared electronic records	Codes: 2 and 5
Saunders (2024) USA	A tool to teach and assess clinical nurse specialist student prescribing competency	The purpose was to assess the usefulness of a tool designed to develop and evaluate clinical nurse specialist (CNS) students' competency and confidence in prescribing therapeutic agents	Of the 18 students, 5 students completed the survey questions at time 1 and 6 students at time 2	The tool in patient care with a supervising mentor during clinical rotations. Surveys after using the tool at 2 points in time	The evaluation tool addressed a gap in assessing prescriber competency among CNSs in real time and provided a means for documentation. Most students reported improved prescribing skills and expressed intent to pursue credentialing and privileging. Further research with larger samples and psychometric validation is recommended to support broader use in CNS education	Codes: 5
Vázquez‐Calatayud et al. (2022) Spain	Nurses' perceptions of the clinical nurse specialist role implemented in a highly specialized university hospital in Spain: A qualitative study	To explore nurses' perceptions of clinical nurse specialist practice as implemented in a highly specialized university hospital in Spain	Seventeen nurses were recruited by purposive sampling	Semi‐structured interviews	Four key categories emerged: role‐holder qualities, practical competencies, team integration, and role impact on nursing, patients, and organizations. The findings support the development and implementation of the clinical nurse specialist (CNS) role, demonstrating its value in enhancing care quality, patient outcomes, and healthcare efficiency	Codes: 4 and 5
Zahran (2013) Jordan	Master's level education in Jordan: A qualitative study of key motivational factors and perceived impact on practice	To explore key motivational factors of Jordanian nurses to undertake a master's degree and explore the perceived impact on practice	Participants (*n* = 37) from five Jordanian hospitals and two public universities	Semi‐structured interviews	Four themes emerged: self‐development, career advancement, practice development, and the perceived impact of master's‐level nurses. Most Jordanian nurses pursued a master's degree to enhance personal and professional growth. M‐level nurses were associated with knowledge transfer, clinical education, and leadership roles. The debate over their impact on patient care extends beyond the Jordanian context	Codes: 1, 3, 4 and 5

*Note:* Publication's role in Results (Code): 1—Novice (and Advanced beginner), 2—Competent, 3—Expert (and Proficient), 4—Task requirements, and 5—Support.

### 2.4 Quality Appraisal

The methodological quality of the included studies was assessed using the Joanna Briggs Institute (JBI) Critical Appraisal Checklists, which are tailored to each study design. These tools were used to evaluate the trustworthiness, relevance, and results of the published studies. The appropriate checklists were selected according to the research method. Study quality was assessed by two researchers (S.I. and M.W.). Initially, both researchers independently assessed the studies, after which the results were compared, and any discrepancies were discussed to reach a consensus. Any uncertainties were further discussed with other members of the research team. Overall, study quality ranged from moderate to good and was considered acceptable, although variation in study design transparency and researcher positioning may influence the interpretation of the findings. Table 3 presents the critical appraisal results.

**TABLE 3:** Quality assessment of included studies using Joanna Briggs Institute (JBI) checklists.

	Q1	Q2	Q3	Q4	Q5	Q6	Q7	Q8	Q9	Q10	Quality assessment
Checklist for quasi‐experimental studies											
	Is it clear in the study what is the ‘cause’ and what is the ‘effect’ (i.e., there is no confusion about which variable comes first)?	Was there a control group?	Were participants included in any comparisons similar?	Were the participants included in any comparisons receiving similar treatment/care, other than the exposure or intervention of interest?	Were there multiple measurements of the outcome, both pre and post the intervention/exposure?	Were the outcomes of participants included in any comparisons measured in the same way?	Were outcomes measured in a reliable way?	Was follow‐up complete and if not, were differences between groups in terms of their follow‐up adequately described and analysed?	Was appropriate statistical analysis used?	—	
Colwill ym. (2014)	N/A	N/A	Y	N	N/A	Y	Y	Y	Y	—	5/6
Saunders (2024)	Y	N/A	Y	N	Y	N	Y	Y	N	—	5/8
Checklist for analytical cross‐sectional studies											
	Were the criteria for inclusion in the sample clearly defined?	Were the study subjects and the setting described in detail?	Was the exposure measured in a valid and reliable way?	Were objective, standard criteria used for measurement of the condition?	Were confounding factors identified?	Were strategies to deal with confounding factors stated?	Were the outcomes measured in a valid and reliable way?	Was appropriate statistical analysis used?	—	—	
Abel and Carter‐Templeton (2020)	Y	N	Y	Y	N	N	Y	Y	—	—	5/8
Doody et al. (2017)	N	Y	Y	Y	N	N	Y	Y	—	—	5/8
Kilpatrick y et al. (2013)	U	Y	Y	Y	N	N	Y	Y	—	—	5/8
Checklist for qualitative research											
	Is there congruity between the stated philosophical perspective and the research methodology?	Is there congruity between the research methodology and the research question or objectives?	Is there congruity between the research methodology and the methods used to collect data?	Is there congruity between the research methodology and the representation and analysis of data?	Is there congruity between the research methodology and the interpretation of results?	Is there a statement locating the researcher culturally or theoretically?	Is the influence of the researcher on the research, and vice‐ versa, addressed?	Are participants, and their voices, adequately represented?	Is the research ethical according to current criteria or, for recent studies, and is there evidence of ethical approval by an appropriate body?	Do the conclusions drawn in the research report flow from the analysis, or interpretation, of the data?	
Fallon et al. (2018)	N	Y	Y	Y	Y	N	N	Y	N	Y	6/10
Fulton et al. (2019)	Y	Y	Y	Y	Y	N	N	Y	N	Y	7/10
Joiner‐Rogers et al. (2019)	N/A	N/A	Y	Y	Y	N	N	Y	N	Y	5/8
Riordan et al. (2019)	Y	Y	Y	Y	Y	N	N	Y	Y	Y	8/10
Vázquez‐Calatayud et al. (2022)	N	Y	Y	Y	Y	Y	Y	Y	Y	Y	9/10
Zahran (2012)	Y	Y	Y	Y	Y	N	N	Y	Y	Y	8/10

*Note:* Y – yes, N – no, U – unclear, N/A – not applicable.

The cut‐off point for the quality appraisal was 50%. The methodological quality varied among the included studies. Nevertheless, no studies were excluded based on the quality assessment. Study selection reliability was assessed using Cohen's kappa to measure agreement between two reviewers. The Cohen's kappa value for title and abstract screening was 0.799, indicating substantial agreement (92.5%). For full‐text selection, the kappa value was 0.507, indicating moderate agreement, with an agreement rate of 87.1% [30].

### 2.5 Data Synthesis

Benner's model ‘From Novice to Expert’ was used as the frame of reference to examine the role of CNSs (especially in the first research question). The theoretical model included a synthesis framework based on the five stages of the model (novice, advanced beginner, competent, proficient, and expert), as illustrated in Figure 1 [22, 23]. Findings from the original studies were extracted, assembled, and categorized according to these stages. A separate data extraction table was used to support the synthesis process, and themes were derived from the data [25]. An example of data integration using a convergent integrated approach is presented in Table 4. In addition, the data also contained descriptions of expertise development that did not align with the stages of Benner's model.

**TABLE 4:** Example of data integration using a convergent integrated approach.

Examples of findings	Categories	Synthesized finding
*Analytical cross‐sectional research*		
	*Received support*	
‘Respondents felt that having **a CNS mentor** (*n* = 18; 60%), a formal transition‐to‐practice program (*n* = 18; 60%), and a standardized job description (*n* = 20; 67%) **would have made a positive difference in their own transitions**.’ (Abel and Carter‐Templeton 2020)	Mentors	Support for professional development from mentors and colleagues
‘…Satisfaction With **Professional Support (5.28/7.0)**. …’ (Abel and Carter‐Templeton 2020)		
*Qualitative research*		
‘Community diabetes nurse specialists, faced with a lack of guidance on their role and a usual ‘safety net’ of resources and other colleagues, **focused on aligning their role with that their peers**. Community diabetes nurse specialists spoke about developing links to compare their role to other diabetes nurse specialists, and to ensure they were delivering their role correctly, or at least in a **similar way to their peers**.’ (Riordan et al. 2019)	Colleagues (professional peer support)	

The data were synthesized using a mixed‐methods review design, integrating both qualitative and quantitative studies through a convergent integrated approach. Quantitative findings were qualitized into textual descriptions, supporting the development of themes and categories for cross‐study comparison and explanation. Integrated findings were derived from these categories. Ensuring transparency and conceptual clarity in the integration process is essential to minimize interpretive bias in mixed‐methods research [31].

Table 2 presents coded information illustrating how each study relates to the theoretical framework and contributes to the synthesis. It indicates the role of each publication in addressing the research questions and supports the interpretation and integration of findings. Research Question 1, ‘How does the expertise of CNSs develop from novice to expert?’, is represented by codes 1–4, based on Benner's theoretical framework, while Research Question 2, ‘How can the development of expertise be supported?’, is represented by code 5. Additionally, a separate data extraction table was created to support the synthesis process, enhancing transparency and traceability in the integration of qualitative and quantitative data.

## 3 Results

### 3.1 Characteristics of Included Studies

The data consisted of 11 research articles describing the development of CNS expertise. The studies focused either entirely or partly on master's‐prepared CNSs or CNS students. The selected research articles were published between 2013 and 2024 and originated from various countries, including the United States [32, 33, 34, 35, 36], Ireland [37, 38, 39], Canada [40], Jordan [41] and Spain [42]. The included studies are presented in Table 2.

### 3.2 Development of Clinical Nurse Specialists' Expertise From Novice to Expert

**FIGURE 3:**
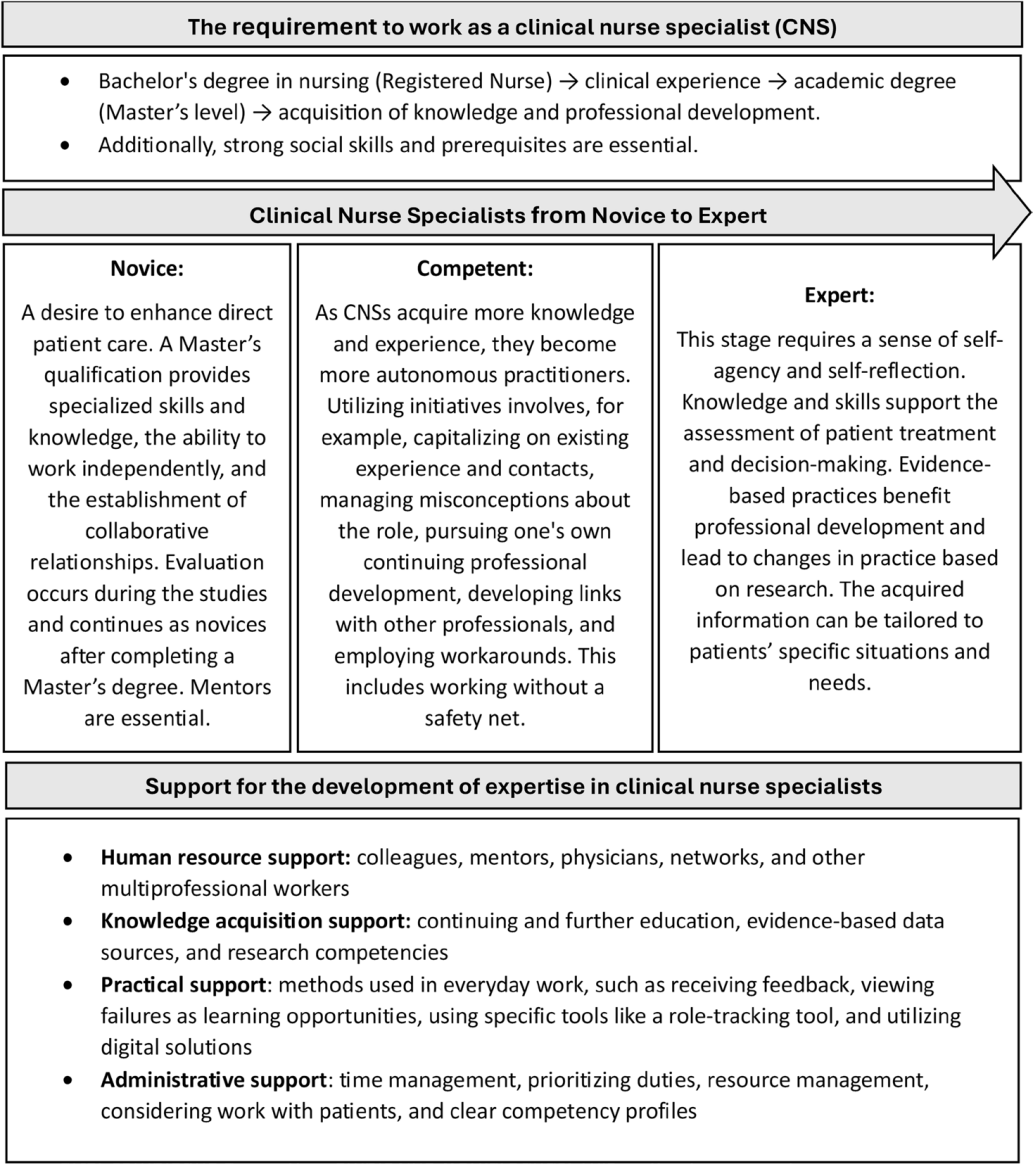
Reflective summary of the results.

To become a CNS, candidates must meet specific requirements. These requirements include a bachelor's degree in nursing [41] and clinical experience [41, 42] after completing a basic degree. In addition, working at an advanced level of nursing requires a master's degree, as well as continuous knowledge development and professional growth [41, 42]. Personal qualities such as an appropriate attitude, dedication, and effective communication skills are essential for nurses working as CNSs. Furthermore, social skills and other personal attributes play a significant role in applying for more advanced tasks [42]. Figure 3 presents both the CNS work requirements and the stages of expertise development from novice to expert among clinical nurse specialists, the latter of which are discussed in more detail in the following sections.

Instead of the five stages of Benner's model [22] (Figure 1), three levels emerged: novice, competent, and expert. In the novice stage, CNSs are motivated to improve direct patient care, which drives the development of professional skills in advanced practice nursing [41]. Registered nurses might reach an expert level in a clinical specialty before entering a master's program, but upon graduation, they become novice CNSs [33]. Students are also considered novices, and the evaluation of their progress begins already during their studies [35]. A master's qualification provides specialized skills, knowledge, and the ability to work independently and establish collaborative relationships [41, 42]. The presence of a CNS mentor positively influences the transition [32].

A competent CNS has more knowledge and experience compared to a novice CNS. At this stage, CNSs also exhibit greater autonomy as practitioners [33, 38]. They can take initiatives, for example, by capitalizing on existing experience and contacts, managing misconceptions about the role, engaging in continuing professional development, developing links with other professionals, and using workarounds. In addition, they can work without relying on a ‘safety net’ [39].

The final stage is the expert CNS, who has a sense of self‐agency expressed through taking responsibility, owning a nursing practice, influencing others, and maintaining self‐awareness through reflection [35]. They have acquired the knowledge and skills to support the assessment of patient treatment and decision‐making [41].

### 3.3 Supporting the Development of Clinical Nurse Specialists' Expertise

The lower section of Figure 3 presents the results supporting the development of CNSs' expertise. In the work of CNSs, expertise develops with the support of colleagues [39], mentors [32], physicians [39, 41], and other multi‐professional workers [35, 39]. Networks play a crucial role in fostering expertise development. In addition, the development of expertise is influenced by further and continuing education [32, 37, 40, 41, 42] and evidence‐based data sources [37, 38]. Development is also supported by methods used in everyday work, such as receiving feedback [37], viewing failures as learning opportunities [35], and using specific tools [33, 36], including role‐tracking tools [33].

Self‐reflection is important for CNSs in professional self‐management. Self‐control is critical for professional performance and project success [36]. CNSs with greater age and experience as registered nurses (RNs) may have increased capacity to focus on nursing practice. Furthermore, greater RN experience is associated with longer tenure in CNS roles, more years at the same institution, and more years of master's‐level nursing education [33].

The development of CNS expertise can be supported by the organization [32, 33, 37, 39, 40, 41], networks [32, 38, 39], increased research competencies [40], reflection of the work with patients [39], and digitalization [34]. The data also described factors that can inhibit the development of expertise. Considering the challenges, the development of expertise can also be supported by time management [33, 38] and prioritization of duties [33, 35, 38]. Additionally, pressures related to resource management [39] were identified.

### 3.4 Summary of the Results

CNS expertise develops through three stages: novice, competent and expert. Qualifications are gained through advanced education, clinical experience and personal qualities. The novice stage involves acquiring foundational competencies with mentor support. In the competent stage, increased knowledge and experience lead to greater independence. In the expert stage, self‐management and professional performance are key. CNS expertise develops continuously, combining experience with new skills. Support from organizations, colleagues, and mentors is crucial. Ongoing learning, often through organizational resources, is necessary to stay updated. Expertise evolves through feedback, learning from failures, and using specific tools. Challenges include time management and task prioritization.

## 4 Discussion

This systematic mixed‐methods review of research evidence (*n* = 11), published between 2013 and 2024, highlights the development of CNS expertise and progression from novice to expert. Benner's model, used as the theoretical framework, was particularly appropriate for the CNS context. The synthesis revealed how individual studies contributed to answering the research questions and aligned with the theoretical framework. This alignment strengthened the coherence of the integrated findings and provided a clearer understanding of how CNS expertise develops and can be supported across different contexts.

The CNS position requires clinical experience and an academic degree obtained after completing a bachelor's degree in nursing. This finding is supported by descriptions of CNSs' work provided by the International Council [1] and national organizations [43, 44], as well as by previous research, including self‐assessments of clinical competence among nursing graduates and the identified need for further education [45]. The development of competence is crucial for attaining expertise, and previous studies have delineated the competencies and roles of CNSs. The clarity of the role is one of the main factors that affect job retention [46]. Competencies should align with the CNS job description and role requirements in response to societal changes [47]. A well‐written job description provides clarity on the role and facilitates efficient work as a CNS [48].

Core competencies reflect the role of CNSs across different countries. In the United States, 44 core competencies are identified across three spheres: direct care of patients, nurses and nursing practice, and organizations and systems [44]. In Canada, core competencies are grouped into four categories: clinical care, system leadership, advancement of nursing practice, and evaluation and research [43]. Similarly, in Finland, four key competency spheres define the role of CNSs: patient care, clinical nursing leadership, organization, and scholarship [3]. The commonalities in CNS competency spheres among these three countries highlight shared objectives. Due to dynamic changes in healthcare environments, responses to population health needs, and CNS practice, competencies for the role will evolve [47].

The review highlights the importance of support mechanisms in the development of CNS expertise. However, as Benner's model does not distinguish between different forms of support, these are presented separately in line with the study aim. Although transitioning to a new role is challenging, mentoring and organizational support can facilitate this process [12, 49]. Expertise is fostered through guidance from mentors, colleagues, and multi‐professional teams, with the need for support diminishing as experience grows and work becomes more efficient. Providing early and ongoing support is crucial to helping CNSs reach competence, benefiting clients and patients through evidence‐based and safe care [39]. Supporting CNSs at both individual and organizational levels is essential, as expertise development requires continuous investment in training and recruitment, with prior clinical experience offering a valuable advantage.

In addition, the results of this review highlighted specific tools and methods that support the assessment of CNS competencies and development. The evaluation of CNSs' expertise takes place during their training [34], with a focus on the development of competence. Therefore, the required competencies should be determined before the assessment of the expertise development [10]. For example, ‘the Competence Validation Checklist’ has been utilized both during the orientation for novices and during the training to become a CNS [50]. The Objective Structured Clinical Examination (OSCE) is another method used to assess competence development [51]. Benner's model has previously been applied to monitor CNS role progression, alongside tools like the PEAC Tool and the CNS Orientation and Development Tool [6, 52]. Core competency scales guide CNS practice and professional growth, facilitating the development of advanced training models based on validated core competencies [53].

The stages of expertise development as conceptualized in Benner's model were illustrated. According to this review, CNSs' expertise develops through three distinct stages: novice, competent and expert. Unlike Benner's five‐stage model [22], this review found no clear distinctions between novices and advanced beginners, or between proficients and experts. This may be attributed to the specific nature of the CNS role, their educational background, and extensive work experience. The merging of these stages aligns with previous understandings, as similar combinations have been made in earlier studies. For instance, McNeil and colleagues developed an assessment tool in which all five of Benner's stages are represented, albeit in a combined form. This tool is designed to support both newly appointed and more experienced CNSs in their annual evaluations [6].

Benner's model describes 2–3 years of experience in the same position to achieve a competent stage of expertise [22]. This review did not identify any specific time frame for this stage, which is characterized by more independent and proactive work, as well as effective work management. Although the required number of years of experience did not become apparent from the data, the expertise of CNSs is developed through experience in the role [41, 42]. Basic knowledge, skills, and competence can be developed and improved alongside clinical and non‐clinical activities [10, 45].

Reaching this competent stage of expertise is undoubtedly helpful when working independently as a CNS. The competent stage may serve as a critical threshold, as reaching this level can significantly influence the CNS's subjective well‐being and overall personal satisfaction. According to this review, CNSs who have reached the competent stage are better able to facilitate their work within the organization [38]. Benner emphasizes the significance of conscious and deliberate planning in this competent stage, fostering efficiency and organizational effectiveness. Consequently, early support and financial resources during a CNS's initial tasks are crucial, as they may contribute to achieving long‐term goals. Acknowledging the time needed for expertise development is pivotal [22]. Progressing through all stages is challenging and requires time for adjustment [9].

According to the findings, digitalization promotes the development of expertise in CNS work [34]. Digitalization and technology facilitate guidance and support for healthcare workers in a remote field environment. Well‐designed systems can improve healthcare availability and outcomes [54], especially in treating chronic diseases and vulnerable groups. Cost savings are achieved, and services become more flexible with the help of digitalization. Communication and remote patient care reduce visits to health services. During training, CNSs are familiarized with methods applied in working life. Future tasks will also emphasize competence in utilizing digital platforms and solutions, managing remote and virtual services, managing and utilizing mobile applications, and developing an open innovation environment [55]. As seen from the findings, it is important to notice that technology and digitalization must also be considered when organizing education for CNSs. Different learning methods should be used to achieve goals and support competencies. In the future, educational harmonization should support graduate programmes through a safe educational tool: a competency profile [53].

The development of CNS expertise is shaped by formal education, continuous professional learning and structured support throughout the career trajectory. Orientation, development plans and role expectations should be tailored to individual needs [6]. Prior clinical experience required for CNS positions promotes and may accelerate expertise development in advanced nursing roles. Nurses themselves have identified advanced training as a key factor enabling CNSs to perform their duties effectively [41]. Experience should guide goal‐setting related to the CNS role, including prioritization within defined time frames [6].

Although this review includes recommendations and requirements for CNS roles, there is no consensus on the educational standards for advanced practice positions. Further education remains essential, but the concept of continuous learning may better capture the ongoing nature of expertise development. While necessary competencies can be achieved, new skills must be acquired continuously to meet evolving clinical demands.

This literature review highlights the advanced expertise of CNSs, which should be more effectively utilized in societal transformation and the development of healthcare service systems. As Kilkku et al. have noted, the advanced practice nurse role offers a meaningful career path for nurses, with several factors positively influencing job satisfaction [46]. Furthermore, evidence‐based practices and CNS education contribute to improved patient care and cost‐effectiveness [21].

These findings align with the Future of Nursing report, which emphasizes the increasing diversity of the CNS role, alongside notable challenges and achievements. To enhance CNS practice and outcomes, policy changes are required [8], and this review reinforces that perspective by underscoring the importance of continuous competence development and collaborative dialogue.

Educational and healthcare organizations play a central role in fostering CNS expertise. Educational institutions should take into account the stages of expertise development and provide appropriate support both during master's‐level education and in continuing professional development. Healthcare organizations contribute by supporting CNSs in clinical practice, which strengthens nursing career paths, recruitment, and retention. Most importantly, expertise development directly enhances CNSs' clinical effectiveness.

These findings are consistent with Benner's theoretical framework, which describes the progression from novice to expert. In addition to identifying developmental stages, this review emphasizes the need to recognize and provide diverse forms of support throughout the professional journey. For educators, this means integrating stage‐specific support into curricula to better prepare CNSs for clinical roles. For healthcare organizations, structured mentorship, role clarity, and clear career development pathways are essential to support CNS growth and retention. Policymakers may consider these findings when designing frameworks that promote advanced practice nursing roles and ensure sustainable workforce development.

### 4.1 Study Limitations and Strengths

Study screening may have involved selection bias, particularly at the title and abstract levels. Thus, despite the two researchers' careful selection process, relevant articles may have been omitted. One of the exclusion criteria was other interventions, such as service development. However, these articles could have provided more perspectives on the development of competence or expertise. The database searches were completed in September 2024. As the manuscript was revised after the initial submission in fall 2024, more recent studies may not have been included. In addition, due to language restrictions, articles in languages other than English and Finnish were not selected. The strengths of this review include consensus in article selection and efforts to minimize bias in examining the theme and phenomenon. Benner's model was suitable for studying the development of CNS expertise. The systematic literature review followed the Joanna Briggs Institute guidelines and was registered with Prospero. Trustworthiness and ethical aspects were addressed throughout all stages of the research.

## 5 Conclusions

This systematic review describes the CNS's progression from novice to expert, beginning when a registered nurse plans a clinical career and pursues further education. Achieving expertise requires clinical experience, master's‐level education, and ongoing professional development, supported by mentorship, employer engagement, and continuous learning, with nurse managers playing a key enabling role. Collaboration between educational institutions and healthcare organizations, along with supportive policies and clear career pathways, is essential. The development of CNS expertise enhances patient care by improving quality, continuity, and effectiveness. These findings inform practice, education, and research, but further research is needed to explore CNS and manager perspectives, organizational support strategies, and the competencies required for future healthcare needs.

## Author Contributions

**Sanna Marika Imeläinen:** conceptualization, methodology, literature search, study selection, quality assessment, data extraction, formal analysis, writing – original draft, writing – review and editing. **Tarja Anneli Kvist:** conceptualization, methodology, study selection, writing – original draft, writing – review and Editing. **Mea Mirella Marjatta Wright:** study selection, quality assessment, writing – original draft, writing – review and editing. **Krista Susanna Jokiniemi:** conceptualization, methodology, study selection, writing – original draft, writing – review and editing. All authors meet the ICMJE criteria for authorship. All authors made substantial contributions to the work, participated in drafting or critically revising the manuscript, approved the final version for publication, and agree to be accountable for all aspects of the work.

## Funding

The authors have nothing to report.

## Ethics Statement

The authors have nothing to report.

## Conflicts of Interest

The authors declare no conflicts of interest.

## Data Availability

The data that support the findings of this study are available from the corresponding author upon reasonable request.
